# High-Speed Multiple Object Tracking Based on Fusion of Intelligent and Real-Time Image Processing

**DOI:** 10.3390/s25113400

**Published:** 2025-05-28

**Authors:** Yuki Kawawaki, Yuji Yamakawa

**Affiliations:** 1 Graduate School of Engineering, The University of Tokyo, Tokyo 153-8505, Japan; 2 Interfaculty Initiative in Information Studies, The University of Tokyo, Tokyo 153-8505, Japan; y-ymkw@iis.u-tokyo.ac.jp

**Keywords:** multiple object tracking, high-speed processing, deep learning, hybrid tracking, multi-processing, tracker management

## Abstract

Multiple object tracking (MOT) is a critical and active research topic in computer vision, serving as a fundamental technique across various application domains such as human–robot interaction, autonomous driving, and surveillance. MOT typically consists of two key components: detection, which produces bounding boxes around objects, and association, which links current detections to existing tracks. Two main approaches have been proposed: one-shot and two-shot methods. While previous works have improved MOT systems in terms of both speed and accuracy, most works have focused primarily on enhancing association performance, often overlooking the impact of accelerating detection. Thus, we propose a high-speed MOT system that balances real-time performance, tracking accuracy, and robustness across diverse environments. Our system comprises two main components: (1) a hybrid tracking framework that integrates low-frequency deep learning-based detection with classical high-speed tracking, and (2) a detection label-based tracker management strategy. We evaluated our system in six scenarios using a high-speed camera and compared its performance against seven state-of-the-art (SOTA) two-shot MOT methods. Our system achieved up to 470 fps when tracking two objects, 243 fps with three objects, and 178 fps with four objects. In terms of tracking accuracy, our system achieved the highest MOTA, IDF1, and HOTA scores with high-accuracy detection. Even with low detection accuracy, it demonstrated the potential of long-term association for high-speed tracking, achieving comparable or better IDF1 scores. We hope that our multi-processing architecture contributes to the advancement of MOT research and serves as a practical and efficient baseline for systems involving multiple asynchronous modules.

## 1. Introduction

Multiple object tracking (MOT) is a crucial and active research topic in computer vision, serving as a fundamental technique across various application domains. These include human–robot interaction [1], autonomous driving [2], surveillance [3], sports video analysis [4], and medical imaging [5]. Robots are increasingly being integrated into human environments, creating a growing demand for systems that enable them to operate effectively in diverse settings and adapt seamlessly to dynamic surroundings. To enhance both robot manipulability and human safety, continuous, accurate, and real-time tracking is essential for downstream tasks like motion planning, reasoning, and collision avoidance [6,7]. Similarly, in autonomous driving, it is critical to detect and track pedestrians emerging from blind spots as early as possible to ensure safety [8]. Thus, an MOT system that balances real-time performance and tracking accuracy is vital for the advancement of these downstream technologies.

MOT is typically approached in two stages: detection, which produces a set of bounding boxes around objects, and association, which links current detections to existing tracks. There are two approaches: one-shot and two-shot methods. One-shot MOT methods unify detection and re-identification (re-ID) within a single network. Previously, it suffered from slow processing time, but FairMOT achieved relatively fast speeds (30 fps) while maintaining tracking accuracy by balancing detection and re-ID tasks [9]. OffsetNet further improved tracking performance and processing speed (50 fps) by integrating amodal bounding box detection, instance segmentation, and tracking into a unified framework [10]. While these one-shot methods achieve strong tracking performance, they still generally fall short of the processing speed offered by geometry-based two-shot approaches.

For online methods, two-shot approaches, tracking-by-detection (TBD), have demonstrated state-of-the-art performance. These methods leverage deep learning-based detectors and employ the Hungarian algorithm [11] for effective inter-frame track assignment. Common association metrics include intersection over union (IoU), Mahalanobis distance, and appearance similarity (e.g., cosine similarity between embedding vectors). To enable real-time processing, lightweight association methods rely primarily on motion and bounding box data. Kalman Filter-based prediction has been widely adopted to track objects in dynamic scenes [12]. However, simple Kalman Filters often struggle with tracker ID switching and are not well suited for long-term tracking. ByteTrack improved association by introducing a two-stage matching strategy [13], while Observation-Centric SORT (OC-SORT) [14] enhanced SORT performance by reducing Kalman Filter error accumulation through virtual trajectory estimation and direction-aware noise modeling. These methods offer a favorable trade-off between accuracy and processing speed among conventional methods. In contrast, some approaches incorporate deep learning-based appearance features to achieve more robust long-term tracking under occlusion, though often at the cost of slower inference speeds [15,16].

Thus, conventional research has advanced MOT systems in terms of processing speed and tracking accuracy. However, current state-of-the-art (SOTA) online methods, such as ByteTrack and OC-SORT, rely on deep learning-based detectors, where the inference time becomes a bottleneck for further speed improvements. To address this, we propose a high-speed MOT system that balances real-time performance, tracking accuracy, and environment-invariant versatility. Our system is composed of two main components: a hybrid tracking framework and a detection label-based tracker management strategy. The hybrid tracking framework integrates three asynchronous modules—deep learning-based detection, classical high-speed detection methods (correlation filters and template matching), and Kalman prediction—to enable high-speed processing and mutually supportive long-term tracking. The detection label-based tracker management synchronously handles multiple trackers across asynchronous threads while maintaining low latency. To further enhance robustness and continuity, we introduce three techniques: dynamic search area adjustment, duplicate tracker elimination, and update skipping for occlusion-aware templates. We evaluate our system in six scenarios using a high-speed camera and compare its performance against current two-shot SOTA methods. The demonstration video is available in [17].

The main contributions of this paper are:

- A holistic multiprocessing algorithm architecture running multiple modules that mutually assist one another.
- High-speed MOT system for multiple categories of objects.
- Efficient detection label-based management of trackers which synchronously handles trackers from asynchronous modules.
- Extensive evaluations of MOT scenarios with a high-speed camera, which have not been worked on.

This paper is organized as follows. Section 2 reviews related work in tracking systems. Section 3 outlines the proposed methods, and effectiveness is demonstrated in Section 4. In Section 5, we evaluate an effect of the individual proposed method. Finally, Section 6 summarizes the proposed method and discusses potential future work.

## 2. Related Work

This section explores two kinds of previous works about tracking methods and Multiple Object Tracking (MOT). Afterward, we provide insights into the strengths and limitations of existing approaches, and we illustrate the novelty of our proposed method.

### 2.1. Tracking Method

We give comprehensive reviews about tracking methods of template image-based, motion-based, deep learning and hybrid methods. For template image-based tracking, template matching [18], calculating the similarity between a pre-defined template image and the current frame, is widely used, but it is not adapted to rotational and scale changes. Additionally, correlation filter-based methods are known for their balancing robustness to scale and rotational changes and processing speed [19,20,21,22,23,24,25]. They employ the fast Fourier transform (FFT) to reduce the computational load required to create and update template images. In particular, the Minimum Output Sum of Squared Error (MOSSE) tracker utilizes raw images to calculate correlation filters, achieving high-speed processing rates of over 500 fps [20]. However, this method is sensitive to changes in appearance, making it less suitable for long-term tracking. While other derivative methods offer higher accuracy, they often sacrifice processing speed. For motion-based tracking, calculating optical flow, which represents the distribution of apparent velocity, is widely used. There are two main methods: feature-based sparse optical flow and pixel-wise dense optical flow [26,27,28]. In sparse optical flow, only selected features are tracked, allowing for high-speed processing. However, finding appropriate and robust features can be challenging. In contrast, some dense optical flow methods, which analyze every pixel, have achieved high-speed processing rates of over 300 fps [27,28]. While it is useful to extract motion of targets with vague boundaries like a human’s joint, it is usually sensitive to background noise. Deep learning-based methods are also employed for versatile tracking applications. For instance, Generic Object Tracking Using Regression Networks (GOTURN), which relies on offline training, achieved tracking of unknown objects at 100 fps [29]. However, GOTURN is sensitive to changes in appearance. Siamese Network-based tracking methods have been widely developed, offering promising results [30,31,32,33,34]. For accurate tracking, the Multi-Domain Convolutional Neural Network (MDNet) combines both offline and online training, but its high computational requirements result in slow processing speed [35]. To achieve a high-speed and robust tracking system, hybrid tracking methods has been tackled. Nishimura et al. [36] achieved robust tracking by integrating several correlation filter-based methods. In [37,38,39,40], a tracking method has been developed that combines deep learning-based detection with template-based tracking, incorporating a retracking mechanism in case the tracking score is low. The work by Matsuo et al. [41] succeeded in tracking a transparent object with low latency (618 fps) combining deep learning-based detection [42], motion detection, and correlation filter-based tracking [20]. Our proposed method was inspired by this research. However, these methods typically focus on a single target, so consistent, efficient management of asynchronous information is required for more complex scenarios such as MOT.

### 2.2. Multiple Object Tracking (MOT)

In general tracking scenarios, the problem of multiple object tracking (MOT) is typically addressed, which involves both detection and association tasks. There are two main types of models: one-shot and two-shot methods.

One-shot methods have addressed challenges such as task conflict between detection and re-identification (re-ID), occlusion handling, and real-time performance. FairMOT [9] achieved 30 fps and improved tracking accuracy by balancing detection and re-ID, extracting features at object centers to avoid the misalignment issue seen in the Joint Detection and Embedding (JDE) model [43]. Occlusion remains a major challenge in MOT. To address this, Trackformer [44] introduced attention mechanisms to enhance tracking robustness, while Preserved ID MOT (PID-MOT) [45] detects appearance changes by predicting object visibility levels and refining features using a visibility-guided association strategy. Furthermore, CSMOT [46] improved re-ID discriminability through angle-center loss and coordinate attention, enhancing small object detection. In terms of real-time performance, OffsetNet (50 fps) [10] unified detection, segmentation, and tracking using pixel-offset representations. Despite these advancements, one-shot methods often remain slower than geometry-based two-shot approaches.

Among two-shot methods, Simple Online and Realtime Tracking (SORT) [12] is a foundational and widely used approach. SORT tracks multiple objects by combining deep learning-based detection with a position-based tracking system using the Kalman Filter and Hungarian algorithm. Although effective, it struggles with irregular motion and frequent identity switches in crowded scenes. Several derivative methods have been proposed. ByteTrack [13] introduced a two-stage matching scheme; BoT-SORT [47] incorporated a revised Kalman Filter and camera motion compensation; OC-SORT [14] carefully reduced Kalman Filter error accumulation. Other methods integrated deep learning-based appearance features, such as DeepSORT [16] and StrongSORT [15], the latter addressing missing detections and associations via the Appearance-Free Link (AFLink) model and Gaussian-Smoothed Interpolation (GSI). BoT-SORT-ReID [47] and Deep OC-SORT [48] have also been proposed. BoostTrack++ [49] is based on BoostTrack [50] by boosting confidence for likely detections and introducing Soft Buffered IoU (BIoU), a similarity measure that combines shape, Mahalanobis distance, and a novel soft IoU metric to refine detection–tracklet association. Improved Association (ImprAssoc) [51] enhanced association performance through a combined matching scheme, integrating distance metrics and occlusion-aware initialization. In summary, two-shot methods have made significant strides in improving both tracking accuracy and processing speed, though much of the focus has been on association techniques rather than accelerating the detection process.

### 2.3. Challenges of Previous Studies

**Figure 1:**
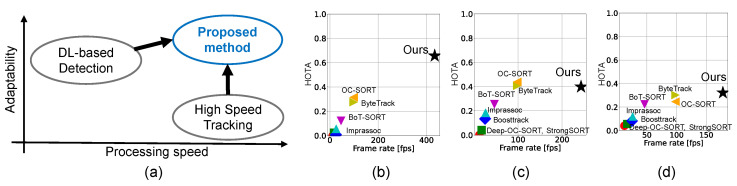
Our motivation and achievements. (**a**) Our motivation. (**b**–**d**) Value of HOTA metric and frame rate for various two-shot tracking methods with (**b**) 2-ball (Section 5.2), (**c**) 3-ball (Section 4.3.4) and (**d**) 4-ball (Section 4.3.5) scenarios.

Previous hybrid approaches primarily focused on simple scenarios, such as single-target tracking. However, in more complex contexts like multiple object tracking (MOT), consistent and efficient management of asynchronous information is essential. In contrast, conventional two-shot MOT systems have mainly prioritized the association process, often placing less emphasis on increasing detection inference speed. As a result, as shown in Table 1, existing systems struggle to meet all key requirements—low latency, high tracking quality, and robustness across diverse environments and target types. To address this, our research aims to develop a versatile tracking system that fulfills all these criteria by accelerating detection and tracker detection label-based tracker management as shown in Figure 1.

## 3. Proposed Methodology

### 3.1. Overview

**Figure 2:**
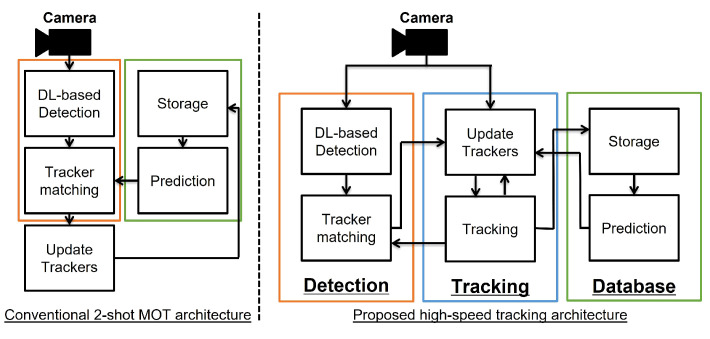
Tracking architecture of the conventional two-shot multiple object tracking and the proposed high-speed tracking systems. The orange box represents the detection module, the blue box denotes the tracking module, and the green box illustrates the database module.

In Figure 2, we compare the proposed architecture with a conventional two-shot MOT system [12]. The conventional system uses geometric predictions to match trackers, processing the entire pipeline sequentially. In contrast, the proposed system runs three threads—detection, tracking, and database—in parallel. This parallel processing allows for faster and more accurate tracking by utilizing not only the predicted values but also high-speed actual tracking data. Additionally, the proposed system detect objects using a deep learning-based method in the detection thread, making our system applicable in a wide range of environments without the need for markers, while maintaining processing speed. This architecture can be extended to systems that aim for efficiency and precision by integrating multiple methods.

**Figure 3:**
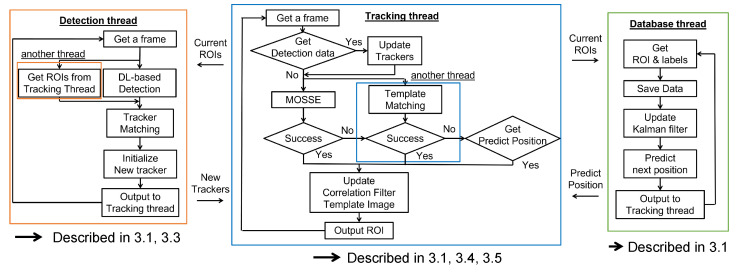
Flowchart of the proposed multiple object tracking system. The orange box represents the detection thread, the blue box represents the tracking thread, and the green box represents the database thread.

Figure 3 illustrates a detailed flowchart of the proposed system. Our approach integrates deep learning-based low-frequency detection, classical high-frequency detection methods—MOSSE and template matching—and Kalman Filter prediction, allowing these components to support each other and achieve robust tracking performance. In the tracking thread, we employ the correlation filter-based method, MOSSE, to handle changes in the target object’s scale and rotation, while template matching offers additional support. Both methods operate concurrently, and their effectiveness is discussed in Section 5.

The tracker’s template image in the tracking thread is updated using results from both the detection thread and the previous step of the tracking thread. For the tracker’s position, we use either the tracking thread or the database thread—based on Kalman Filter prediction, which offers faster updates than deep learning-based detection. In the database thread, Kalman Filter models with constant velocity assumptions are updated using results from the tracking thread. Further details on data exchange and tracker management are provided in Section 3.2.

### 3.2. Detection Label-Based Tracker Management

We describe how multiple trackers are managed based on detection labels.

First, we introduce the structure of each tracker and the types of data exchanged between threads. The state of each tracker is modeled as $\left\{\mathrm{label},x,y,\dot{x},\dot{y},w,h,\mathrm{CF},\mathrm{template},\mathrm{trackingScore}\right\}$. Here, label is the detection label; *x* and *y* represent the center position of the ROI; $\dot{x}$ and $\dot{y}$ represent the velocity; *w* and *h* denote the width and height of the ROI; CF represents the correlation filter used in MOSSE; template refers to the template image; and trackingScore indicates the score from each tracking method, including the Peak-to-Sidelobe Ratio (PSR) and Zero-mean Normalized Cross-Correlation (ZNCC).

Next, we explain the communication among the detection, tracking, and database threads. Exchanges between the detection and tracking threads include template images and their corresponding bounding box information. Details of this communication are explained in Section 3.3 and Section 3.4. Concerning exchanges between tracking and databased threads, the tracking thread sends updated tracker information—such as the latest geometric data and a list of trackers marked for removal—to the database thread, which uses this data to update its Kalman Filter models. When each Kalman Filter is sufficiently updated (i.e., the update count reaches ${\mathrm{Counter}}_{\mathrm{valid}}$), the database thread returns predicted positions to the tracking thread.

**Figure 4:**
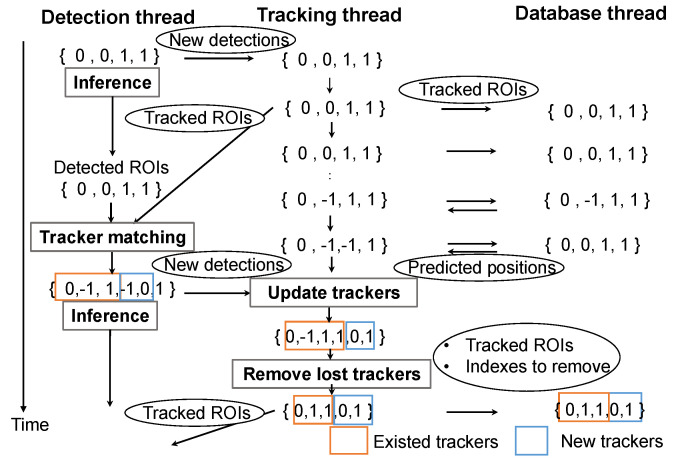
Detection label-based tracker management. {0,0,1,1} represents a list of detection labels. The arrows indicate data flow among the detection, tracking, and database threads. Rectangular boxes denote processes, while circular nodes represent exchanged data. Orange boxes indicate existing trackers, and blue boxes indicate new trackers detected by the detection thread.

We illustrate the detection label-based tracker management process with a simple example in Figure 4. The indexing rule is straightforward; non-negative indices (e.g., ‘0’ and ‘1’) represent valid detection labels, while lost trackers are indexed as ‘−1’. The tracker container preserves the order of trackers throughout the system to ensure consistent and synchronous tracking.

We now explain how the tracker container is synchronized and updated. To synchronize the next tracker candidates between the detection and tracking threads, as soon as the tracking thread updates tracker information based on detection results, it sends the latest tracker data to the detection thread. This information is first passed through a buffer thread to avoid interfering with deep learning inference.

Tracker removal is handled carefully to maintain consistency across threads. If a tracker has not been updated for several frames (i.e., exceeds $Counter_{\mathrm{lost}}$), it is marked for removal. Since the number of trackers should only change when new deep learning-based detection results are available, removal occurs only when the tracking thread receives new tracker data from the detection thread. Trackers marked for deletion are removed when the tracking thread sends the latest tracker list to the detection and database threads, as illustrated in the “Remove lost trackers” part of Figure 4. Trackers with label ‘−’ in the orange box are removed from both the tracking and detection threads in this example. As mentioned above, to keep the database thread synchronized, the tracking thread sends not only the updated ROI information but also the indices of the removed trackers.

This detection label-based management strategy enables our MOT system to track objects efficiently with low latency, regardless of object type or quantity.

### 3.3. Tracker Matching

**Figure 5:**
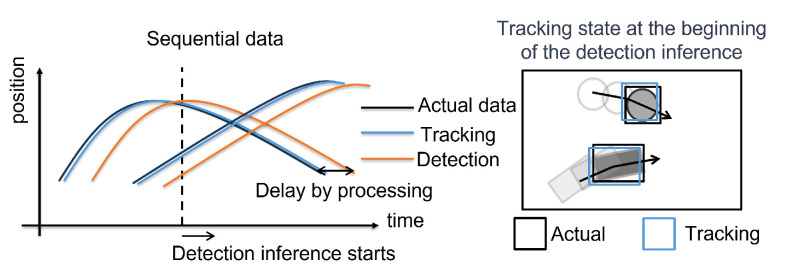
(**left**) shows sequential data; the black line represents the ground truth, the blue line indicates the tracked data from the tracking thread, and the orange line shows the detection data from the detection thread. The horizontal offsets between the blue and orange lines relative to the black line illustrate the delays caused by processing time. The vertical dashed line marks the moment when deep learning-based detection inference begins. (**right**) illustrates the tracking state corresponding to the time indicated by the dashed line in the left figure.

The matching process between trackers in the detection and tracking threads is illustrated in Figure 5, which shows sequential data and the tracking state at the beginning of deep learning-based detection inference. As depicted, the tracking thread operates with low latency, resulting in minimal deviation between the actual and tracked positions. Therefore, by using the most recent tracked ROIs available in the tracking thread, the proposed system can accurately associate trackers between the detection and tracking threads.

To perform tracker matching, we apply the Hungarian algorithm [11], with the cost defined as follows in Equation (1):

(1) $$\begin{array}{cc} cost_{\mathrm{label}} & =\left\{\begin{array}{cc} 0 & \mathrm{if}\;\mathrm{detection}\;\mathrm{labels}\;\mathrm{are}\;\mathrm{the}\;\mathrm{same}\\ {\mathrm{Cost}}_{\max} & \mathrm{otherwise} \end{array}\right.\\ cost_{\mathrm{IoU}} & =\left\{\begin{array}{cc} \frac{1}{\mathrm{IoU}} & \mathrm{if}\;\mathrm{IoU}>\frac{1}{{\mathrm{Cost}}_{\max}}\\ {\mathrm{Cost}}_{\max} & \mathrm{otherwise} \end{array}\right.\\ cost_{\mathrm{total}} & =cost_{\mathrm{label}}+cost_{\mathrm{IoU}} \end{array}$$

The total cost is the sum of the label-based cost and the cost derived from the overlap between bounding boxes, calculated using Intersection over Union (IoU).

In conventional methods [12,13,14], tracker matching typically relies on predicted positions from Kalman Filters, which are effective only in scenarios with simple motion. In contrast, the proposed method improves matching accuracy by utilizing real-time tracking data from the tracking thread.

### 3.4. Hybrid Tracking

**Figure 6:**
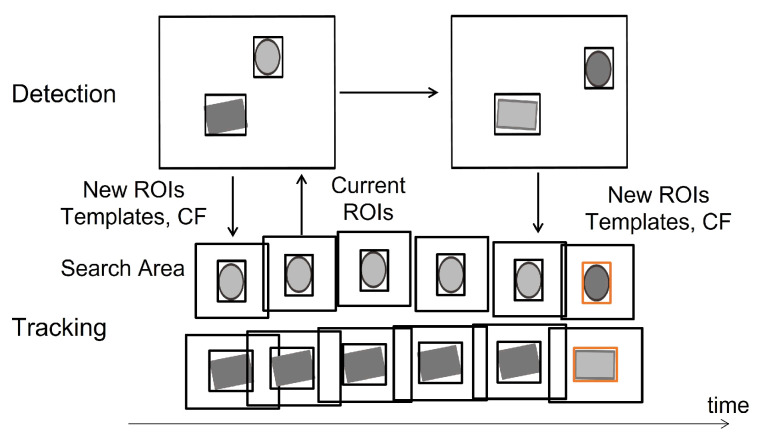
Overview of the hybrid tracking system combining deep learning-based low-frequency detection with classical high-frequency tracking methods. In the detection row, the large black box represents the entire image, while the smaller boxes around the objects indicate their ROIs. In the tracking row, the larger black boxes denote the search areas, and the smaller boxes represent the ROIs of the tracked objects. The horizontal axis indicates the time sequence. Orange boxes highlight the moments when the templates and correlation filters in the tracking thread are updated based on detection results.

Figure 6 illustrates our hybrid tracking architecture inspired from [41]. New correlation filters (CFs) and templates from the detection thread are used to update the trackers only if the tracking score from the tracking thread is under threshold.

To effectively define the search area around the target object when tracking is successful, the size of the search region, ($w_{\mathrm{search}},h_{\mathrm{search}}$), is adaptively adjusted based on the velocity of the tracked object, as defined in Equation (2). In the case of the first detection or when the tracker has not been updated for more than 100 ms, both $scale_{X}$ and $scale_{Y}$ are set to the maximum value, ${\mathrm{scale}}_{\max}$.

(2) $$\begin{array}{cc} w_{\mathrm{search}} & =scale_{X}\cdot w_{\mathrm{ROI}}\\ h_{\mathrm{search}} & =scale_{Y}\cdot h_{\mathrm{ROI}}\\ scale_{X} & =\max\left(\min\left(1+\frac{\Delta x}{w_{\mathrm{ROI}}},{\mathrm{scale}}_{\max}\right),{\mathrm{scale}}_{\min}\right)\\ scale_{Y} & =\max\left(\min\left(1+\frac{\Delta y}{h_{\mathrm{ROI}}},{\mathrm{scale}}_{\max}\right),{\mathrm{scale}}_{\min}\right) \end{array}$$

Here, ($w_{\mathrm{ROI}},h_{\mathrm{ROI}}$) represent the current ROI’s size, and Δx and Δy denote the horizontal and vertical movements from the previous detection, respectively. Both $scale_{X}$ and $scale_{Y}$ are constrained within the range [${\mathrm{scale}}_{\min}$ (=1.5), ${\mathrm{scale}}_{\max}$ (=3.0)].

### 3.5. Handling Trackers

Methods for handling duplicate trackers and short-term occlusion are described in this section.

In crowded scenarios, multiple trackers may point to the same object, leading to redundant computations and increased processing time due to the larger number of trackers. To address this, we perform duplicate checking every time the tracking thread receives new data from the detection thread, when new trackers are likely to be generated. This procedure is based on two metrics: Intersection over Union (IoU) and velocity similarity. If both conditions in Equation (3) are satisfied, the redundant tracker is removed:

(3) $$\begin{array}{cc} \mathrm{IoU} & >{\mathrm{IoU}}_{\mathrm{duplication}}\\ \frac{v_{i}\cdot v_{j}}{|v_{i}\left|\cdot \right|v_{j}|} & >\cos(\theta_{\mathrm{threshold}}) \end{array}$$

Here, ${\mathrm{IoU}}_{\mathrm{duplication}}$ is the threshold for determining duplication, and $v_{i}$ and $v_{j}$ denote the velocity of the respective trackers. The second condition in Equation (3) evaluates the directional similarity between the two trackers using the cosine of the angle between their velocity vectors.

When removing duplicate trackers, the system retains the tracker with the higher tracking score in terms of templates and correlation filters. As for the tracker itself, the one with the longer lifespan is preserved. This update strategy enables efficient tracking and contributes to extending the effective lifespan of valid trackers.

Secondly, to address short-term occlusions, we skip updating the correlation filter (CF) and template images in the tracking thread. During occlusion, we expect to see a discontinuity in the tracking score and movement. Therefore, if a tracker detects one of the discontinuities defined in Equation (4), the CF and template image are not updated. Instead, we only update the tracker’s position based on Kalman Filter prediction.

(4) $$\begin{array}{cc} \Delta \mathrm{trackingScore} & \geq \Delta {\mathrm{trackingScore}}_{\mathrm{threshold}}\\ \frac{v_{t-1}\cdot v_{t}}{|v_{t-1}\left|\cdot \right|v_{t}|} & \leq \cos(\theta_{\mathrm{threshold}}) \end{array}$$

Here, ΔtrackingScore represents the change in tracking score, $\Delta {\mathrm{trackingScore}}_{\mathrm{threshold}}$ represents the threshold for this change, $v_{t-1}$ and $v_{t}$ denote the velocity at times (t−1) and *t*, respectively, and $\cos(\theta_{\mathrm{threshold}})$ represents the threshold for changes in direction. The first condition assesses an abrupt appearance change, and the second condition assesses an abrupt direction change. The consecutive skipping is up to ${\mathrm{Count}}_{skipping}$.

## 4. Experiment

The demonstration video of our experiments can be found here [17].

### 4.1. Settings

We conducted experiments using a camera (XIMEA, Münster, Germany) set to 400 fps. Since the proposed method relies on classical high-speed detection and requires a high-frame-rate video, we collected our own dataset rather than using a public one. We used a PC as follows:

- CPU: AMD Ryzen 5 7645HX with Radeon Graphics;
- GPU: NVIDIA GeForce RTX 4050 Laptop GPU.

**Figure 7:**
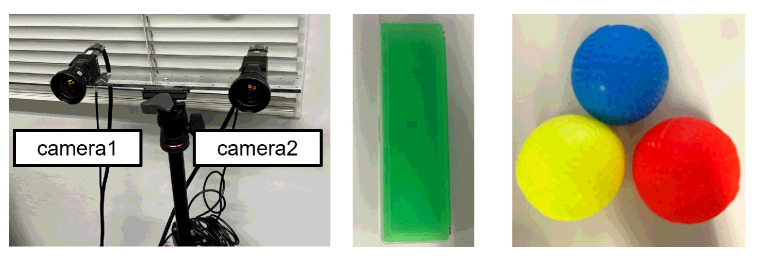
Experiment settings. (**left**) illustrates the cameras used to capture videos. (**right**) illustrates objects, plastic case and balls.

For all results presented in this paper, we compare each method and perform offline analyses. The experimental settings are illustrated in Figure 7.

### 4.2. YOLO Training

For multiple object tracking, we used the YOLOv8n model [52] for detections and trained it with a customized dataset, which includes a plastic box and balls, as depicted in Figure 7. We used 6500 images for training. We used the YOLOv8-x detector for reference data.

### 4.3. Multiple Object Tracking

We conducted five tracking experiments for 2 objects (2 accelerated crossing objects, 2 categories, including a plastic case and ball, thrown balls), 3 objects, and 4 objects. The purposes are as follows:

- Two accelerated crossing balls: Evaluate tracking accuracy during abrupt motion changes and assess prevention of tracker switching.
- Two categories (plastic box and ball): Check whether the system is applicable to multi-category tracking and robust to rotational variations.
- Thrown balls: Assess the system’s adaptability to changes in object scale and background conditions.
- Three and four balls: Evaluate the system’s capability to track more than two objects, tracking accuracy in dense environments, and robustness to complete occlusion.

The hyperparameters used for this comparison are listed in Table 2.

We compared the proposed system with seven state-of-the-art tracking modules implemented based on BoxMOT [53]. From the slowest to the fastest in terms of processing speed, the tracking modules include Deep OC-SORT [48], StrongSORT [15], BoostTrack++ [49], Imprassoc [51], BoT-SORT [47], ByteTrack [13], and OC-SORT [14]. All tracking methods used a pretrained YOLOv8n model for detection. Except for BoT-SORT, ByteTrack, and OC-SORT, we employed a Re-ID model based on the Omni-Scale Network (OSNet) [54] to extract appearance features.

We evaluated tracking accuracy using five metrics: Intersection over Union (IoU), mean Average Recall (mAR, Equation (5)) mean Average Precision (mAP, Equation (6)), Multiple Object Tracking Accuracy (MOTA, Equation (7)) [55], IDF1 (Equation (8)) [56], and Higher Order Tracking Accuracy (HOTA, Equation (9)) [57] including Detection Accuracy (DetA) and Association Accuracy (AssA).

(5) $$\mathrm{mAR}=\frac{\left|\mathrm{TP}\right|}{\left|\mathrm{TP}|+|\mathrm{FN}\right|}$$

(6) $$\mathrm{mAP}=\frac{\left|\mathrm{TP}\right|}{\left|\mathrm{TP}|+|\mathrm{FP}\right|}$$

(7) $$\mathrm{MOTA}=1-\frac{\left|\mathrm{FN}|+|\mathrm{FP}|+|\mathrm{IDSW}\right|}{\left|\mathrm{gtDet}\right|}$$

(8) $$\mathrm{IDF}1=\frac{\left|\mathrm{IDTP}\right|}{\left|\mathrm{IDTP}|+0.5\cdot |\mathrm{IDFN}|+0.5\cdot |\mathrm{IDFP}\right|}$$

(9) $$\begin{array}{cc} \mathrm{HOTA} & =\frac{1}{19}\cdot \Sigma_{\alpha \in \{0.05,\ldots ,0.95\}}{\mathrm{HOTA}}_{\alpha }\\ {\mathrm{HOTA}}_{\alpha } & =\sqrt{{\mathrm{DetA}}_{\alpha }\cdot {\mathrm{AssA}}_{\alpha }}\\ \mathrm{DetA} & =\frac{\left|\mathrm{TP}\right|}{\left|\mathrm{TP}|+|\mathrm{FN}|+|\mathrm{FP}\right|}\\ \mathrm{AssA} & =\frac{\Sigma_{c\in \mathrm{TP}}\mathrm{Ass}-\mathrm{IoU}\left(c\right)}{\left|\mathrm{TP}\right|}\\ \mathrm{Ass}-\mathrm{IoU} & =\frac{\left|TPA\right|}{\left|TPA|+|FNA|+|FPA\right|} \end{array}$$

Here, |TP|, |IDTP|, and |TPA| represent the number of true positive trackers; |FN|, |IDFN|, and |FNA| indicate the number of false negative trackers; |FP|, |IDFP|, and |FPA| denote the number of false positive trackers; and |gtDet| is the number of ground truth trackers. |IDSW| indicates the number of tracker ID switches between consecutive frames, and α denotes the IoU threshold for detection, ranging from 0.05 to 0.95 in increments of 0.05. In our setting, MOTA, IDF1, and HOTA are averaged over all α values.

To calculate mAP, MOTA, and DetA, we performed frame-level matching between the predicted and ground truth trackers using the IoU and the Hungarian algorithm. For IDF1 and AssA, tracker pairs were determined by applying the Hungarian algorithm to minimize the total number of false positive and false negative trackers across all possible associations.

#### 4.3.1. Tracking of 2 Accelerated Balls

**Figure 8:**
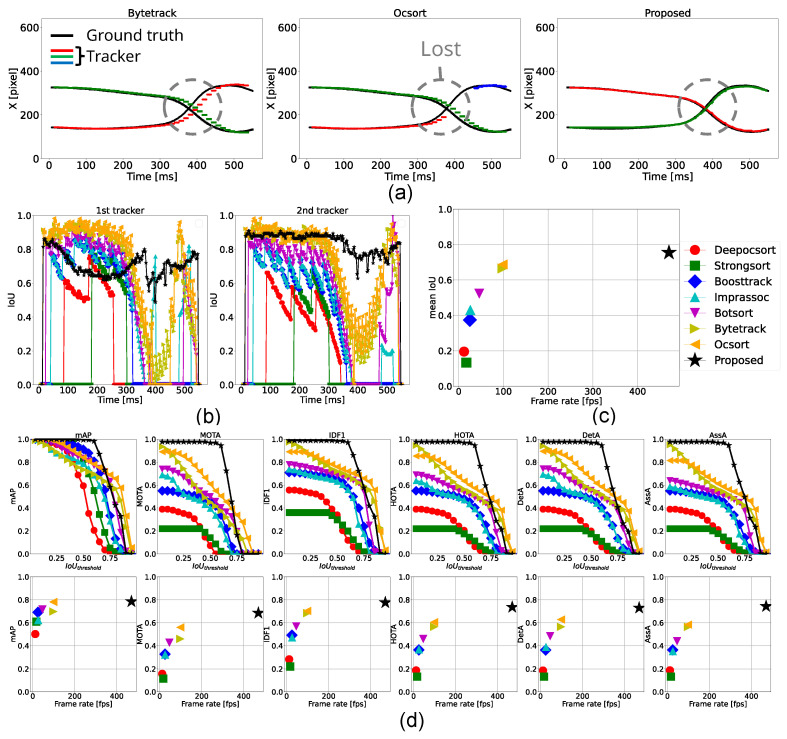
Tracking results for two accelerated balls. (**a**) Trajectories of the horizontal position (x-coordinate) of the ball centers tracked by ByteTrack, OC-SORT, and the proposed method. Plots with colors, but black represents each estimated trackers. (**b**) Temporal transition of IoU for each ground-truth tracker. (**c**) Comparison of each method in terms of frame rate and mean IoU. (**d**) Top row: Relationship between IoU threshold and performance metrics including mAP, MOTA, IDF1, HOTA, DetA, and AssA. Bottom row: Relationship between frame rate and each performance metric.

According to Table 3, the proposed method operated at 470 fps, which was over 4.7 times faster than existing methods, and outperformed all conventional approaches across all metrics for both detection and association accuracy. As shown in the estimated center positions and IoU transitions in Figure 8a,b, when the two balls began accelerating around 300 ms, the proposed method was able to track both objects with high precision and lower latency compared to ByteTrack and OC-SORT. As a result, OC-SORT lost track of the objects when the two balls crossed and one ball was partially occluded. These results demonstrate that the proposed method is robust to abrupt motion changes and partial occlusions and capable of accurately tracking fast-moving objects.

On the other hand, in the metric transitions shown in Figure 8d, the proposed method exhibited slightly lower scores than ByteTrack and OC-SORT at higher IoU thresholds. This drop was primarily due to false detections from the classical detector, which was observed in the first tracker from around t = 100∼300 ms in Figure 8, which deviated from the ground truth. This implied that the proposed method requires frequent updates by deep learning-based detections due to its limited robustness in long-term tracking.

Moreover, the bottom panels of Figure 8c,d show that processing speed has a more significant impact than deep learning-based appearance features on both detection and association performance.

#### 4.3.2. Two Categories of Object Tracking

According to Table 4, the proposed method operated faster and outperformed all conventional approaches across all metrics in ball tracking. However, for box tracking, BoT-SORT, ByteTrack, and OC-SORT achieved better performance in both detection and association. This discrepancy was primarily due to the proposed method’s lower detection accuracy and its inability to adapt to dynamically changing object shapes. One main factor was the reduced inference speed of deep learning-based detectors when multiple modules were executed simultaneously.

**Figure 9:**
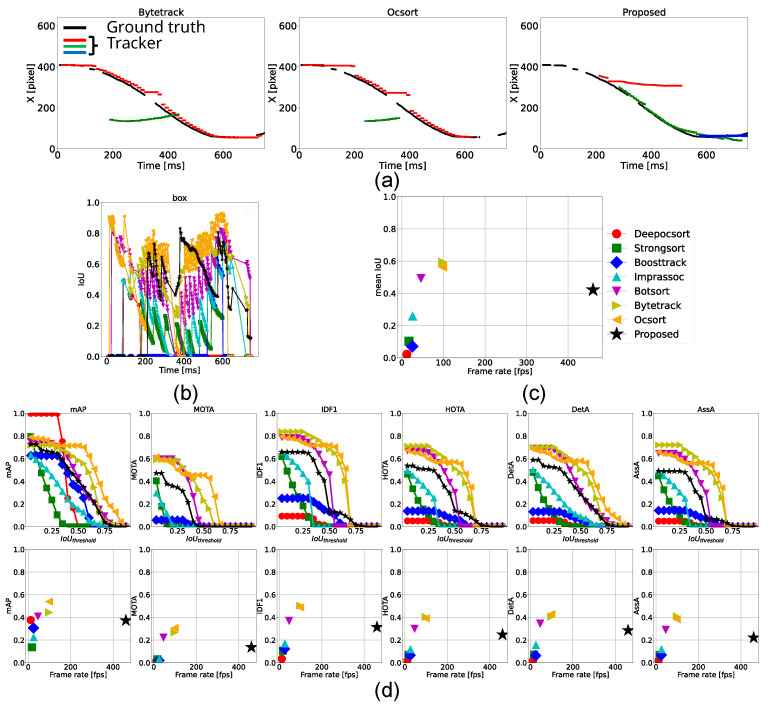
Tracking results for box tracking. (**a**) Trajectories of the horizontal position (x-coordinate) of the ball centers tracked by ByteTrack, OC-SORT, and the proposed method. (**b**) Temporal transition of IoU for each ground-truth tracker. (**c**) Comparison of each method in terms of frame rate and mean IoU. (**d**) Top row: Relationship between IoU threshold and performance metrics. Bottom row: Relationship between frame rate and each performance metric.

As shown by the red plots in Figure 9a, ByteTrack and OC-SORT successfully tracked the box, while the red and blue plots from the proposed method failed to do so. Notably, around t=300 ms, the YOLOv8 model failed to detect the box, resulting in gaps in the black plots. In contrast, the green plots from the proposed method interpolated the missing data and continued tracking. However, during this period, the tracker was unable to accurately adjust the bounding box shape, leading to a drop in IoU, as shown in Figure 9b.

Similarly, in the experiment with two accelerated balls, Figure 9c,d demonstrated a clear positive correlation between processing speed and tracking performance metrics.

In conclusion, this experiment confirmed that the proposed tracking system is capable of handling multi-category objects. However, for non-circular objects, high-frequency tracker updates using robust detection methods are essential. As future work, we plan to improve long-term robustness in tracking non-circular objects by both accelerating deep learning-based detection and developing a fast and rotation-invariant detection method.

#### 4.3.3. Tracking of 2 Thrown Balls

**Figure 10:**
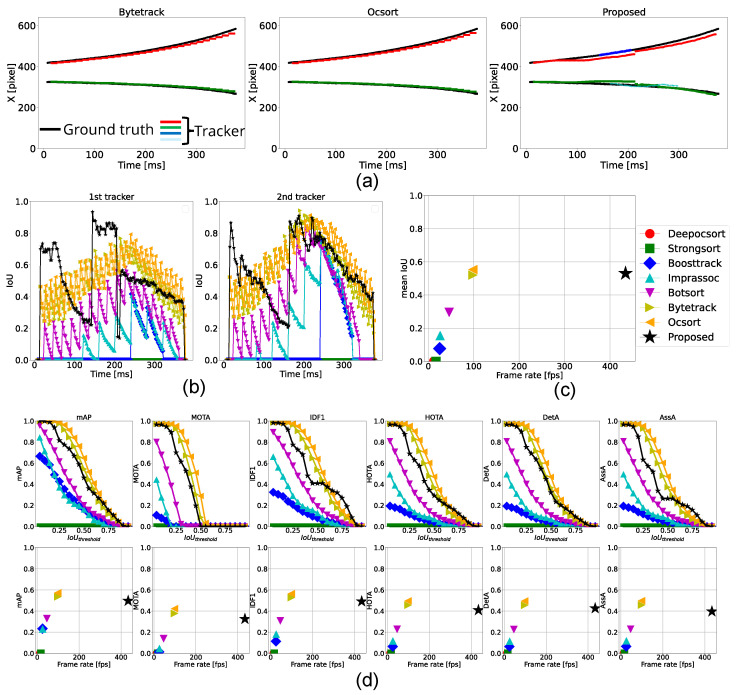
Tracking results for two thrown balls. (**a**) Trajectories of the horizontal position (x-coordinate) of the ball centers tracked by ByteTrack, OC-SORT, and the proposed method. (**b**) Temporal transition of IoU for each ground-truth tracker. (**c**) Comparison of each method in terms of frame rate and mean IoU. (**d**) Top row: Relationship between IoU threshold and performance metrics. Bottom row: Relationship between frame rate and each performance metric.

According to Table 5, the proposed method operated faster; however, OC-SORT achieved higher values across all metrics. To understand the underlying cause, we refer to Figure 10. As shown by the red and green plots in Figure 10a, ByteTrack, OC-SORT, and the proposed method consistently tracked the same objects. However, the tracker in the proposed method slightly deviated from the ground truth center positions, which led to the initiation of additional trackers, visualized with blue and cyan plots. As seen in the IoU jump around t=200 ms in Figure 10b, these duplicate trackers were eventually merged with the original ones through the duplication handling process described in Section 3.5. In the metric calculations, however, these duplicate trackers were counted as false positives in both detection and association, resulting in lower overall scores. To address this limitation, using coverage over the smaller bounding box instead of IoU may offer a more effective criterion. Nevertheless, the duplication-check procedure must be carefully designed to balance precision and recall, especially in crowded environments. Resolving this trade-off remains an important direction for future work.

Considering the low scores of slow-processing methods in Table 5 and the effect of processing speed on tracking metrics shown in Figure 10c,d, we conclude that high-speed tracking is essential for real-time recognition in dynamic environments.

This experiment demonstrated that the proposed method is adaptable to changes in object scale and background. However, it still faces challenges in managing duplicate trackers considering the precision–recall trade-off as well as ensuring robust and high-frequency tracker updates.

#### 4.3.4. Tracking of 3 Balls

**Figure 11:**
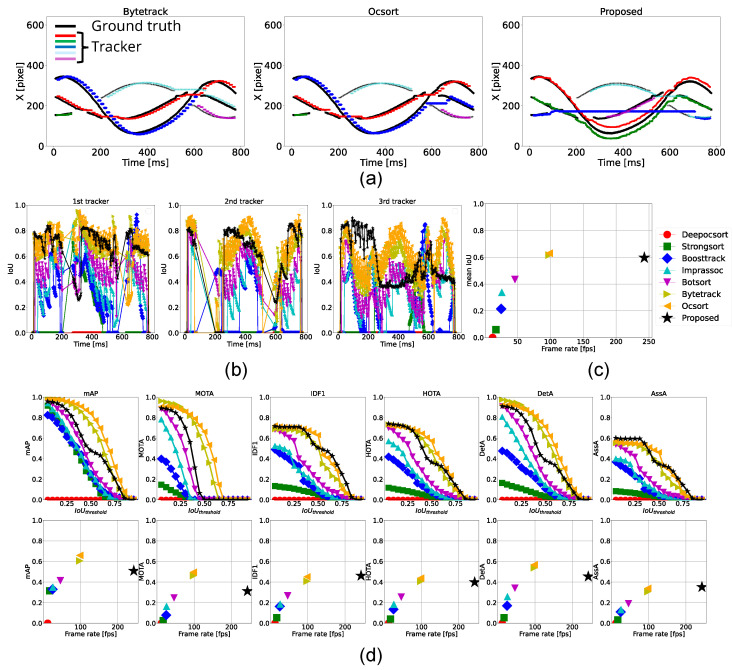
Tracking results for three balls. (**a**) Trajectories of the horizontal position (x-coordinate) of the ball centers tracked by ByteTrack, OC-SORT, and the proposed method. (**b**) Temporal transition of IoU for each ground-truth tracker. (**c**) Comparison of each method in terms of frame rate and mean IoU. (**d**) Top row: Relationship between IoU threshold and performance metrics. Bottom row: Relationship between frame rate and each performance metric.

According to Table 6, the proposed method operated at 243 fps, which is over 2.5 times faster than the conventional methods. ByteTrack and OC-SORT achieved higher scores in detection-related metrics such as mAP, MOTA, and DetA, while the proposed method outperformed them in association metrics, including IDF1 and AssA. As shown in Figure 11a, the blue plots from ByteTrack and OC-SORT and the red plots from the proposed method represent the foremost ball. Among these, only the proposed method continuously tracked the same ball throughout the sequence, indicating its ability of continuous and respective tracking in crowded environments. However, similar to conventional methods, the proposed method still faced challenges in handling complete occlusions and duplicate trackers. To address complete occlusions, leveraging the classical high-speed detection method not only for fine-grained tracking but also for occlusion detection could be effective. Detecting occlusions through a drop in tracking scores can prevent trackers from switching to other objects, allowing them instead to rely on Kalman Filter predictions to wait for the occluded object to reappear. Alternatively, deploying a multi-camera system or extending to 3D tracking are promising options for improvement, which will be explored in future work.

As depicted in the figures, tracker switching in Figure 11a and lower association scores in Figure 11c,d, particularly in crowded environments, increased latency caused by slower processing speeds leads to greater deviations from the actual object positions. It results in an increase in the likelihood of tracker ID switches. Therefore, maintaining high processing speed is essential for accurate and consistent tracking.

This experiment demonstrated that the proposed method is capable of tracking more than two objects and that high-speed tracking significantly enhances discrimination and robustness in crowded environments.

#### 4.3.5. Tracking of 4 Balls

According to Table 7, the proposed method operated at 178 fps, which is over 1.8 times faster than the conventional methods. In this experiment, a similar trend to the three-ball tracking case was observed; ByteTrack and OC-SORT achieved higher scores in detection-related metrics such as mAP, MOTA, and DetA, while the proposed method outperformed them in association metrics, including IDF1 and AssA. Notably, the proposed method also achieved the highest score in HOTA.

**Figure 12:**
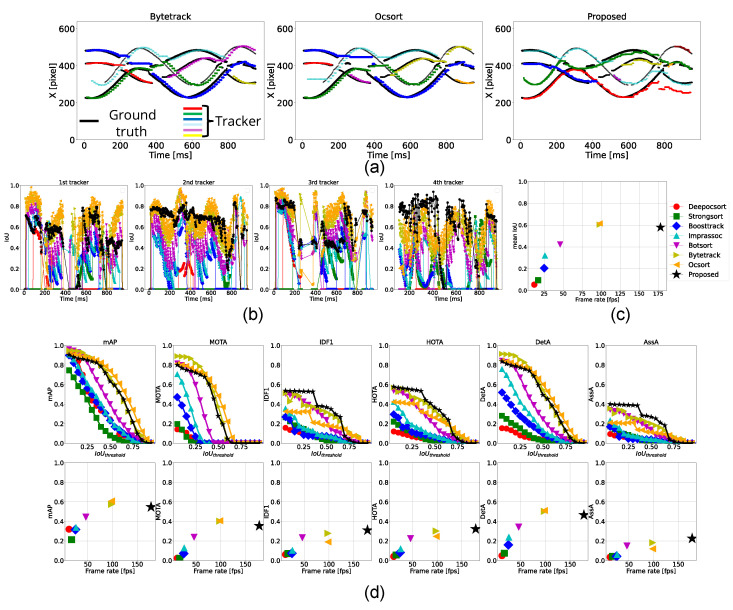
Tracking results for four balls. (**a**) Trajectories of the horizontal position (x-coordinate) of the ball centers tracked by ByteTrack, OC-SORT, and the proposed method. (**b**) Temporal transition of IoU for each ground-truth tracker. (**c**) Comparison of each method in terms of frame rate and mean IoU. (**d**) Top row: Relationship between IoU threshold and performance metrics. Bottom row: Relationship between frame rate and each performance metric.

As shown in Figure 12a, the green plots from ByteTrack and OC-SORT and the red plots from the proposed method represent the foremost ball. None of the methods maintained consistent tracking throughout, but the proposed method successfully handled the first crossing, where the conventional methods failed. However, from t=700 ms, the proposed method lost the ball. This was caused because the tracker was prevented from updating by the deep learning-based module because its tracking score exceeded the threshold, as described in Section 3.4. Since the frame-level (local) tracking score does not always ensure consistent object identity, incorporating a global metric to evaluate tracking consistency is necessary. One potential solution is a temporal trajectory-aware metric, such as the state variance of a Kalman Filter.

As depicted in the tracker switching results in Figure 12c,d, detection-related scores such as mean IoU, mAP, MOTA, and DetA exhibited an exponential saturation behavior, while association-related scores like IDF1, AssA, and HOTA showed a monotonically increasing trend. In the proposed method, detection accuracy primarily depends on deep learning-based detection, while association accuracy is attributed to the hybrid tracking mechanism. Assuming both scores follow similar trends with respect to processing speed, it is inferred that while deep learning-based detection degraded at high speeds, the hybrid tracker compensated for poor detections using classical high-speed tracking methods.

In summary, we verified that the proposed method runs faster than conventional methods when tracking four objects simultaneously. We also demonstrated the effectiveness of hybrid tracking in improving association performance and confirmed the necessity of enhancing detection accuracy to achieve balanced overall tracking performance.

#### 4.3.6. Summary of Tracking Experiments

We summarize the results of the five experiments in Table 8. Through these experiments, we confirmed that the proposed method achieved high-speed processing—up to 4.7× faster (470 fps) for two objects, 2.5× faster (243 fps) for three objects, and 1.8× faster (178 fps) for four objects, while maintaining versatility in terms of working environments, target types, and object quantities. The proposed method demonstrated superior performance across all metrics in the two-accelerated-ball experiment. However, it usually suffered from lower detection-related accuracy due to reduced deep learning-based detection performance caused by simultaneous multi-threaded operations. Despite this, our method showed better association performance than the conventional method, as indicated by metrics such as IDF1 and association accuracy (AssA). Therefore, by enhancing detection accuracy as shown in Table 8, the proposed system can achieve precise long-term tracking.

Furthermore, by comparing processing speed with each evaluation metric, ByteTrack and OC-SORT consistently achieved the best performance in MOTA, IDF1, and HOTA across all scenarios. We therefore conclude that in dynamically changing environments and when using a high-speed camera, processing speed has a more significant impact than deep learning-based appearance features on both detection and association performance.

Lastly, we briefly estimate the maximum objects where our method operates faster than the conventional methods. The processing speed of our system is primarily determined by the tracking time, $time_{tracking}$, of MOSSE and template matching, as preprocessing and postprocessing each take less than 50 μs. In our setup, $time_{tracking}$ per iteration was approximately 1.3 ms, based on averaged experimental processing time. Due to CPU core limitations, we applied MOSSE and template matching to each tracker sequentially rather than simultaneously. Therefore, assuming memory bandwidth is not a limiting factor, our system can operate faster than conventional methods when tracking seven or fewer objects.

## 5. Ablation Study

### 5.1. Effectiveness of Each Method

We confirmed the effects of each element of our proposed methods with a two-ball-crossing situation. The methods to be checked are as follows:

1. Tracking thread: Correlation filter-based tracking, MOSSE.
2. Tracking thread: Template matching.
3. Tracking thread: MOSSE + Template matching.
4. Database thread (Section 3.2): Compensation by Kalman prediction.
5. How to update trackers (Section 3.4): Check tracking score of the current templates.
6. Dynamic search area size adjustment based on velocity of trackers (Equation (2)).
7. Delete duplicate trackers (Section 3.5).
8. Skip updating templates (Section 3.5).

From now on, the indices greater than 3 represent methods that build upon the previously listed methods by incorporating the technique corresponding to the given index. For example, Method 5 represents one combining techniques 3, 4 and 5. Additionally, for methods prior to Method 6, the search area size is fixed at three times the size of the current ROI. We evaluated the contribution of each method in terms of processing speed and tracking accuracy.

**Figure 13:**
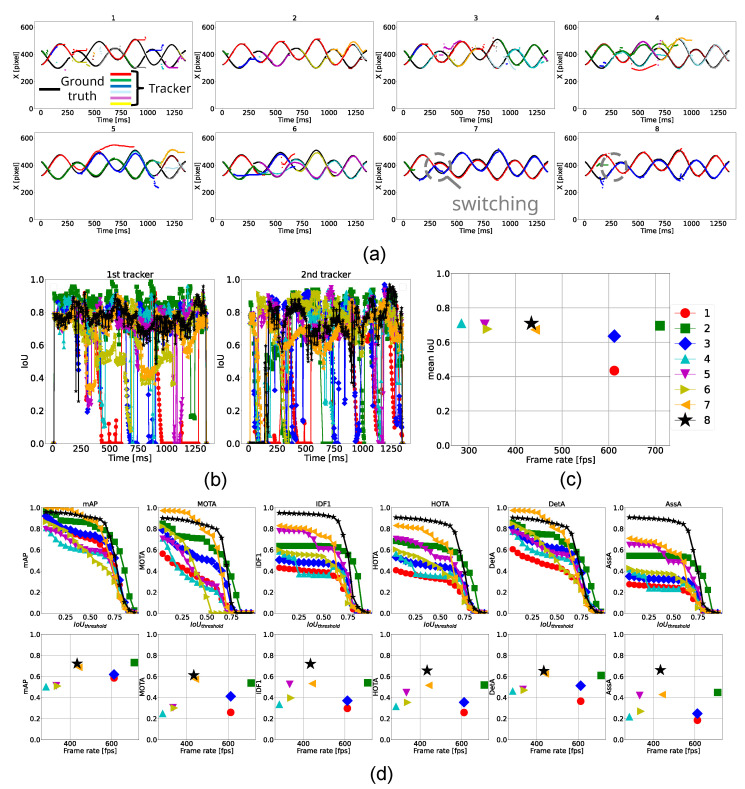
Tracking results for two balls. (**a**) Trajectories of the horizontal position (x-coordinate) of the ball centers tracked by 8 methods. (**b**) Temporal transition of IoU for each ground-truth tracker. (**c**) Comparison of each method in terms of frame rate and mean IoU. (**d**) Top row: Relationship between IoU threshold and performance metrics. Bottom row: Relationship between frame rate and each performance metric.

The results are presented in Table 9. First, we compared Methods 1 to 3: “MOSSE”, “Template Matching”, and “MOSSE + Template Matching”. Among these, Method 2 achieved the best performance in both tracking accuracy and processing speed, with a MOTA of 0.538, IDF1 of 0.539, and HOTA of 0.52. However, as shown in the top-left three panels of Figure 13a, multiple colors appear within the same object, indicating that none of these methods could handle long-term tracking. For subsequent methods, we adopt Method 3 (“MOSSE + Template Matching”), as MOSSE is known for its robustness to scale and rotation variations.

Comparing Method 4 with Method 3 in Table 9, the integration of Kalman prediction enables the system to maintain object tracking over a longer duration and achieves the best average IoU. In the top-right panel of Figure 13a, black-colored plots are not visible except during *t* = 50∼200 ms, where other colors appear, indicating an improved metric, recall. This can be confirmed its highest score in mAR at 0.723 from Table 9. However, the number of trackers assigned to the same ball increased, resulting in degraded MOTA, IDF1, HOTA, and processing speed.

In Method 5, a tracking-score-based update procedure was introduced to prioritize the use of recent, high-confidence templates over obsolete deep learning-based ones. As shown in the bottom-left panel of Figure 13a, the length of the same-colored plots increased compared to Method 4, suggesting more consistent tracking and a decrease in redundant trackers. Consequently, as confirmed in Table 9, processing speed slightly improved while maintaining appropriate templates. However, we still see some duplicate trackers remaining, and performance in metrics other than average IoU was still lower than the best-performing method (Method 2).

According to the results of Method 6 in Table 9, dynamically adjusting the search area size slightly improved the processing speed to 340 fps but did not enhance tracking performance. Nevertheless, for other scenarios, we adopted this function to enable efficient exploration by reducing unnecessary searches.

In Method 7, as shown in Table 9 and the seventh panel of Figure 13a, removing duplicate trackers significantly improved the processing speed to 442 fps and extended tracker lifespan by merging duplicate trackers. As a result, Table 9 showed that Method 7 achieved the highest MOTA score of 0.574 and competitive IDF1 and HOTA scores of 0.532 and 0.516, respectively. As shown in the top panels of Figure 13d, all metrics for Method 7 exceeded those of Method 2 at an ${\mathrm{IoU}}_{\mathrm{threshold}}$ of 0.5. However, at higher ${\mathrm{IoU}}_{\mathrm{threshold}}$ values, Method 2 achieved better scores, resulting in comparable overall performance in IDF1 and HOTA.

In the eighth row of Figure 13a, the introduction of update skipping successfully prevented a tracker ID switch around t=300 ms, an issue unresolved in Method 7. Furthermore, consistent color plots in Figure 13a, stable IoU values exceeding 0.6 in Figure 13b, and the highest scores in each metric by ${\mathrm{IoU}}_{\mathrm{threshold}}=0.75$ shown in Figure 13b indicate that both balls were reliably tracked in terms of detection and association. Although the tracker occasionally deviated from the ground truth around *t* = 900 ms, as shown in Figure 13a, due to update skipping, it successfully recovered to the correct position through deep learning-based updates. As shown in Table 9, Method 8 (our proposed method) achieved the best overall performance, with a MOTA of 0.611, IDF1 of 0.719, and HOTA of 0.656.

Finally, the contributions of each component are summarized in Table 10.

### 5.2. Comparison with Other Methods

**Figure 14:**
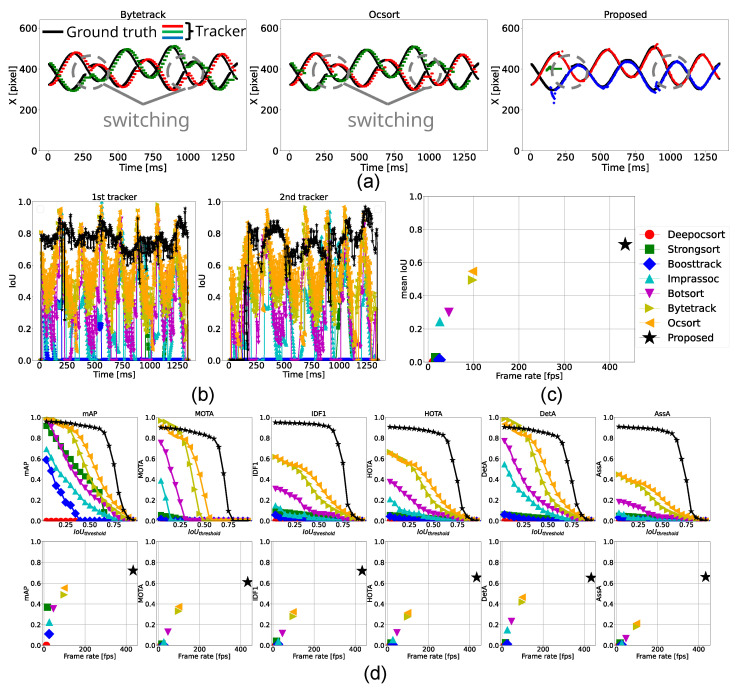
Tracking results for two balls. (**a**) Trajectories of the horizontal position (x-coordinate) of the ball centers tracked by ByteTrack, OC-SORT, and the proposed method. (**b**) Temporal transition of IoU for each ground-truth tracker. (**c**) Comparison of each method in terms of frame rate and mean IoU. (**d**) Top row: Relationship between IoU threshold and performance metrics. Bottom row: Relationship between frame rate and each performance metric.

According to Table 11, the proposed method operated faster than existing methods, and outperformed all conventional approaches across all metrics for both detection and association accuracy. As shown in the estimated center positions in Figure 14a, ByteTrack and OC-SORT suffered from tracker ID switching around t=300 ms and t=1000 ms, while the proposed method overcame this. As shown in IoU and metric transitions in Figure 14b–d, the proposed method kept tracking with higher IoU, resulting in higher metrics over all ${\mathrm{IoU}}_{\mathrm{threshold}}$. These results demonstrate that the proposed method is effective in handling partial occlusions and long-term fast object tracking. As in other scenarios, the bottom panels of Figure 14c,d show that processing speed has greater influence than deep learning-based appearance features on both detection and association performance.

## 6. Conclusions

Conventional tracking systems have struggled to satisfy all the essential criteria for a versatile tracking solution: low latency, high accuracy, and robustness across diverse environments and varying numbers and types of targets. To address this, we proposed a high-speed multiple object tracking (MOT) system that meets all of these requirements.

Our system is mainly composed of two components. The first is a hybrid tracking framework that integrates deep learning-based detection, correlation filter tracking, template matching-based detection, and Kalman prediction for motion compensation. The second is a detection label-based tracker management strategy that synchronously handles multiple trackers across asynchronous threads while maintaining low latency. To ensure robust and continuous tracking, we further proposed three techniques: dynamic search area adjustment, duplicate tracker elimination, and update skipping for occlusion-aware templates. These methods’ effectiveness was verified through ablation studies.

We compared our system with seven conventional methods across six scenarios using recent MOT evaluation metrics, including IoU, mAR, mAP, MOTA, IDF1, and HOTA. Since our system leverages classical high-speed detection, it requires a high-speed camera—an aspect rarely addressed in prior work. Across all scenarios, we observed that in dynamically changing environments and when using a high-speed camera, processing speed has a more significant impact than deep learning-based appearance features on both detection and association performance.

Our experiments demonstrated the effectiveness of our system as follows. In terms of processing speed, we achieved up to 4.7× faster operation (470 fps) for two objects, 2.5× faster (243 fps) for three objects, and 1.8× faster (178 fps) for four objects. In the two-ball accelerated motion scenario and two-ball crossing motion, our system consistently achieved the best performance across all metrics. It also proved robust to partial occlusions and successfully mitigated tracker ID switches, which conventional methods struggled to overcome. In experiments involving two object categories (ball and box) and two thrown balls, our system demonstrated robustness to multiple categories and changes in rotation, scale, and background. However, conventional methods such as ByteTrack and OC-SORT outperformed our system in MOTA, IDF1, and HOTA under some conditions, largely due to the relatively lower detection accuracy in our framework. In three- and four-object tracking scenarios, our system showed strong performance, particularly in IDF1 and association accuracy (AssA), highlighting its ability to maintain robust associations despite lower detection accuracy.

While our system achieved comparable or superior tracking accuracy, it has three limitations and presents two key challenges for future work. First, as the system relies on classical high-speed detection, its effectiveness is demonstrated only with a high-frame-rate video. Second, to improve long-term accuracy for non-circular objects, we plan to enhance detection performance by accelerating deep learning-based methods and developing high-speed, rotation- and scale-invariant detection algorithms. Third, to increase robustness against occlusion, we will implement fine-grained occlusion detection using global and local context-aware tracking metrics, and extend the system to support multi-camera tracking.

Finally, we introduced a multi-processing architecture that demonstrated effectiveness for high-speed MOT. We hope that our proposed system contributes to further advancements in MOT and serves as an efficient baseline for systems that integrate multiple asynchronous modules.

## Figures and Tables

**Table 1:** Comparison between conventional methods and the proposed method. ✓ represents “good quality”.

Method	Low Latency	Accuracy	Environments	Types and Number of Targets
Hybrid method [41]	✓	✓	✓	
One-shot method [9,10]		✓	✓	✓
Two-shot method [13,14]		✓	✓	✓
Proposed Method	✓	✓	✓	✓

**Table 2:** Hyperparameters in the proposed MOT system.

Parameter		Value
Tracker lifespan	${\mathrm{Counter}}_{\mathrm{valid}}$	4
	${\mathrm{Counter}}_{\mathrm{lost}}$	5
Tracking score	${\mathrm{PSR}}_{\min}$	5.0
	${\mathrm{ZNCC}}_{\min}$	0.0
Duplicate Trackers (Equation (3))	${\mathrm{IoU}}_{\mathrm{duplication}}$	0.6
Skip updating (Equation (4))	$\Delta {\mathrm{trackingScore}}_{\mathrm{threshold}}$	2.0
	$\cos(\theta_{\mathrm{threshold}})$	π/2
	${\mathrm{Count}}_{\mathrm{skipping}}$	3

**Table 3:** Tracking results for two accelerated balls with mean IoU, mAR, mAP, MOTA, IDF1, HOTA, DetA and AssA. The bold value indicates the best score for each metric.

Method	Frame Rate (fps)	IoU			mAR	mAP	MOTA	IDF1	HOTA	DetA	AssA
		**1st**	**2nd**	**Average**							
deepocsort	12	0.184	0.206	0.195	0.196	0.502	0.157	0.282	0.187	0.186	0.188
strongsort	17	0.149	0.118	0.133	0.134	0.611	0.116	0.22	0.132	0.132	0.133
boosttrack	25	0.381	0.368	0.374	0.381	0.692	0.327	0.492	0.363	0.362	0.364
imprassoc	26	0.467	0.394	0.43	0.434	0.623	0.322	0.472	0.368	0.389	0.352
botsort	46	0.51	0.537	0.523	0.532	0.716	0.427	0.57	0.46	0.484	0.439
bytetrack	98	0.65	0.68	0.665	0.673	0.698	0.46	0.684	0.565	0.565	0.565
ocsort	98	0.665	0.71	0.688	0.699	0.782	0.56	0.704	0.605	0.628	0.584
proposed	**470**	**0.686**	**0.823**	**0.755**	**0.767**	**0.785**	**0.685**	**0.776**	**0.736**	**0.73**	**0.744**

**Table 4:** Tracking results for ball and box tracking. The data are presented in the format of “ball’s metric/box’s metric”. The bold value indicates the best score for each metric.

Method	Frame Rate (fps)	IoU	mAR	mAP	MOTA	IDF1	HOTA	DetA	AssA
deepocsort	12	0.01/0.021	0.007/0.02	0.06/0.377	0.002/0.018	0.012/0.035	0.007/0.019	0.007/0.02	0.007/0.018
strongsort	17	0.065/0.103	0.062/0.094	0.256/0.137	0.041/0.031	0.1/0.107	0.059/0.071	0.059/0.073	0.059/0.069
boosttrack	25	0.131/0.071	0.126/0.07	0.334/0.307	0.091/0.02	0.183/0.123	0.118/0.066	0.118/0.064	0.118/0.069
imprassoc	26	0.254/0.26	0.251/0.254	0.424/0.225	0.172/0.034	0.252/0.171	0.193/0.12	0.224/0.158	0.18/0.119
botsort	46	0.341/0.493	0.334/0.496	0.345/0.409	0.157/0.222	0.339/0.37	0.253/0.3	0.254/0.346	0.253/0.291
bytetrack	98	0.57/**0.594**	0.575/**0.6**	0.589/0.445	0.42/0.268	0.581/**0.504**	0.5/**0.404**	0.501/0.407	0.5/**0.409**
ocsort	98	0.609/0.563	0.615/0.571	0.63/**0.539**	0.451/**0.308**	0.622/0.489	0.538/0.391	0.538/**0.43**	0.537/0.384
proposed	**460**	**0.675**/0.42	**0.684**/0.423	**0.691**/0.373	**0.583**/0.137	**0.686**/0.314	**0.632**/0.248	**0.633**/0.286	**0.631**/0.222

**Table 5:** Tracking results for two thrown balls. The bold value indicates the best score for each metric.

Method	Frame Rate (fps)	IoU			mAR	mAP	MOTA	IDF1	HOTA	DetA	AssA
		**1st**	**2nd**	**Average**							
deepocsort	12	0	0	0	0	0	0	0	0	0	0
strongsort	17	0	0	0	0	0	0	0	0	0	0
boosttrack	25	0.049	0.105	0.077	0.075	0.234	0.013	0.114	0.065	0.064	0.065
imprassoc	26	0.102	0.209	0.156	0.149	0.231	0.045	0.181	0.116	0.115	0.118
botsort	46	0.247	0.344	0.295	0.288	0.329	0.139	0.308	0.227	0.226	0.229
bytetrack	98	0.473	0.567	0.52	0.521	0.535	0.378	0.528	0.456	0.455	0.457
ocsort	98	0.505	**0.601**	**0.553**	**0.557**	**0.573**	**0.423**	**0.565**	**0.497**	**0.495**	**0.498**
proposed	**434**	**0.508**	0.553	0.53	0.531	0.497	0.326	0.492	0.409	0.427	0.397

**Table 6:** Tracking results for three balls. The bold value indicates the best score for each metric.

Method	Frame Rate (fps)	IoU				mAR	mAP	MOTA	IDF1	HOTA	DetA	AssA
		**1st**	**2nd**	**3rd**	**Average**							
deepocsort	12	0	0	0	0	0	0	0	0	0	0	0
strongsort	17	0.129	0.001	0.042	0.059	0.057	0.316	0.026	0.054	0.041	0.054	0.032
boosttrack	25	0.335	0.158	0.156	0.215	0.212	0.33	0.079	0.163	0.135	0.17	0.109
imprassoc	26	0.408	0.285	0.319	0.33	0.3338	0.351	0.164	0.186	0.184	0.259	0.132
botsort	46	0.503	0.434	0.377	0.434	0.432	0.414	0.247	0.267	0.255	0.342	0.191
bytetrack	98	**0.698**	**0.623**	0.544	0.617	0.624	0.604	0.462	0.409	0.407	0.54	0.308
ocsort	98	0.695	0.599	**0.6**	**0.63**	**0.639**	**0.658**	**0.497**	0.453	**0.439**	**0.567**	0.341
proposed	**243**	0.668	0.596	0.538	0.597	0.604	0.51	0.312	**0.462**	0.399	0.455	**0.352**

**Table 7:** Tracking results for four balls. The bold value indicates the best score for each metric.

Method	Frame Rate (fps)	IoU					mAR	mAP	MOTA	IDF1	HOTA	DetA	AssA
		**1st**	**2nd**	**3rd**	**4th**	**Average**							
deepocsort	12	0.063	0.032	0.095	0.033	0.053	0.052	0.32	0.024	0.062	0.041	0.049	0.034
strongsort	17	0.088	0.004	0.168	0.142	0.094	0.09	0.214	0.022	0.071	0.056	0.075	0.043
boosttrack	25	0.231	0.188	0.304	0.112	0.205	0.2	0.316	0.072	0.074	0.082	0.16	0.043
imprassoc	26	0.323	0.361	0.426	0.184	0.321	0.315	0.338	0.129	0.104	0.12	0.238	0.061
botsort	46	0.401	0.441	0.565	0.304	0.422	0.421	0.444	0.239	0.233	0.224	0.341	0.148
bytetrack	98	**0.586**	0.625	0.717	0.503	0.604	0.61	0.575	0.402	0.278	0.302	0.503	0.183
ocsort	98	0.557	**0.661**	**0.717**	0.538	**0.615**	**0.623**	**0.61**	**0.407**	0.19	0.246	**0.511**	0.12
proposed	**178**	0.469	0.654	0.604	**0.591**	0.579	0.586	0.548	0.355	**0.31**	**0.322**	0.466	**0.226**

**Table 8:** Summary of the five MOT experiments.

Experiment	Frame Rate (fps)	Achievements	Challenges and Future Work
2 Accelerated Balls (Section 4.3.1)	470	Robust to abrupt motion changes and partial occlusions, capable of accurately tracking fast-moving objects.	Requires high-frequency updates from deep learning-based detections for robust long-term tracking.
2 Categories (Section 4.3.2)	460	Capable of handling multi-category objects.	Improve tracking of non-circular objects by accelerating deep learning-based detection and developing a fast, rotation-invariant detection method.
2 Thrown Balls (Section 4.3.3)	434	Adaptable to changes in object scale and background.	Manage duplicate trackers while balancing the precision–recall trade-off, ensuring robust and high-frequency tracker updates.
3 Balls (Section 4.3.4)	243	Capable of tracking more than two objects with higher association accuracy.	Address complete occlusion by implementing fine-grained tracking-based occlusion detection and expanding to a multi-camera system.
4 Balls (Section 4.3.5)	178	Demonstrates the effectiveness of hybrid tracking in improving association performance.	Combine frame-level local tracking with trajectory-aware global scores to achieve long-term tracking.

**Table 9:** Tracking results for two balls. The bold value indicates the best score for each metric.

Method	Frame Rate (fps)	IoU			mAR	mAP	MOTA	IDF1	HOTA	DetA	AssA
		**1st**	**2nd**	**Average**							
1	612	0.459	0.409	0.435	0.44	0.586	0.259	0.297	0.258	0.365	0.184
2	**710**	**0.812**	0.57	0.696	0.709	**0.733**	0.538	0.539	0.52	0.609	0.448
3	612	0.678	0.59	0.636	0.645	0.619	0.411	0.371	0.355	0.513	0.248
4	284	0.748	0.67	**0.711**	**0.723**	0.503	0.248	0.335	0.316	0.461	0.218
5	334	0.691	0.717	0.703	0.715	0.512	0.304	0.525	0.446	0.477	0.419
6	340	0.614	**0.745**	0.677	0.686	0.509	0.301	0.396	0.354	0.47	0.27
7	442	0.689	0.655	0.673	0.682	0.687	0.574	0.532	0.516	0.624	0.428
8	434	0.753	0.662	0.71	**0.723**	0.722	**0.611**	**0.719**	**0.656**	**0.653**	**0.661**

**Table 10:** Effects of each proposed MOT technique on processing speed and tracking accuracy, recall, precision and association. The upward arrow indicates an improvement in the score, while the downward arrow indicates a deterioration.

Method	Processing Speed	Recall	Precision	Association
MOSSE + Template matching	-	↑	↑	↑
Kalman Filter	↓	↑	-	-
Check tracking scores	-	-	↑	↑
Dynamic search area size adjustment	↑	-	-	-
Delete duplicate trackers	↑	-	↑	↑
Skip updating trackers	-	-	-	↑

**Table 11:** Tracking results for two balls. The bold value indicates the best score for each metric.

Method	Frame Rate (fps)	IoU			mAR	mAP	MOTA	IDF1	HOTA	DetA	AssA
		**1st**	**2nd**	**Average**							
deepocsort	12	0	0	0	0	0	0	0	0	0	0
strongsort	17	0.007	0.046	0.026	0.026	0.369	0.011	0.041	0.023	0.025	0.022
boosttrack	25	0.022	0.004	0.013	0.012	0.11	0.001	0.01	0.007	0.011	0.005
imprassoc	26	0.252	0.236	0.244	0.237	0.224	0.033	0.039	0.055	0.15	0.021
botsort	46	0.305	0.294	0.3	0.295	0.354	0.129	0.115	0.122	0.231	0.065
bytetrack	98	0.496	0.494	0.495	0.495	0.489	0.331	0.279	0.275	0.418	0.182
ocsort	98	0.569	0.523	0.547	0.551	0.555	0.374	0.324	0.315	0.465	0.214
proposed	**434**	**0.753**	**0.662**	**0.71**	**0.723**	**0.722**	**0.611**	**0.719**	**0.656**	**0.653**	**0.661**

## Data Availability

The original contributions presented in this study are included in the article. Further inquiries can be directed to the corresponding author.
